# Toward automated plantar pressure analysis: machine learning-based segmentation and key point detection across multicenter data

**DOI:** 10.3389/fbioe.2025.1579072

**Published:** 2025-06-19

**Authors:** Carlo Dindorf, Jonas Dully, Steven Simon, Dennis Perchthaler, Stephan Becker, Hannah Ehmann, Christian Diers, Christoph Garth, Michael Fröhlich

**Affiliations:** ^1^ Department of Sports Science, University of Kaiserslautern-Landau (RPTU), Kaiserslautern, Germany; ^2^ DIERS International GmbH, Wisebaden, Germany; ^3^ Department of Computer Science, University of Kaiserslautern-Landau (RPTU), Kaiserslautern, Germany

**Keywords:** artificial intelligence, deep learning, image segmentation, zoning, intelligent systems, biomechanics, U-net, hallux angle

## Abstract

Plantar pressure analysis is a pivotal tool for assessing foot function, diagnosing deformities, and characterizing gait patterns. Traditional proportion-based segmentation methods are often limited, particularly for atypical foot structures and low-quality data. Although recent advances in machine learning (ML) offer opportunities for automated and robust segmentation across diverse datasets, existing models primarily rely on data from single laboratories, limiting their applicability to multicenter datasets. Furthermore, the prediction of relevant landmarks on the plantar pressure profile has not been explored. This study addresses these gaps by exploring ML-based approaches for anatomical zone segmentation and landmark detection in plantar pressure analysis, including 758 plantar pressure samples from 460 individuals (197 females, 263 males) collected from multiple centers during static and dynamic conditions using two distinct systems. The datasets were further standardized and augmented. The plantar surface was segmented into four regions (hallux, metatarsal area 1, metatarsal areas 2–5, and the heel) using a U-Net model, and deep learning regression models predicted the key points, such as interdigital space coordinates and the center of metatarsal area 1. The results underscore the U-Net’s capacity to attain an accuracy comparable to that of experts (Median Dice Scores ≥ 0.88), particularly in regions with well-defined plantar pressure boundaries. Metatarsal area 1 exhibited unique characteristics because of its ambiguous boundaries, with expert reviews playing a valuable role in enhancing accuracy in critical cases. Using a regression model (Median Euclidean distance = 7.72) or an ensemble model (Median Euclidean distance = 5.26) did not improve calculating the center of metatarsal area 1 directly from the segmentation model (Median Euclidean distance = 4.47). Furthermore, regression-based approaches generated higher errors in key point detection of the interdigital space 2–3 (Median Euclidean distance = 10.06) than in metatarsal area 1 center (Median Euclidean distance = 7.72). These findings emphasize the robustness of the proposed segmentation and key point prediction models across diverse datasets and hardware setups. Overall, the proposed methods facilitate the efficient processing of large, multicenter datasets across diverse hardware setups, significantly reducing the reliance on extensive human labeling, lowering costs, and minimizing subjective bias through ML-driven standardization. Leveraging these strengths, this work introduces a novel framework that integrates multicenter plantar pressure data for both segmentation and landmark detection, offering practical value in clinical and research settings by enabling standardized, automated analyses across varying hardware configurations.

## 1 Introduction

Foot deformities represent a common problem among the different age groups and sexes in Western societies (Spahn et al., 2004; Bogut et al., 2019; Chua et al., 2021) and can cause injuries to the lower limbs or even back pain (Chuter and Janse de Jonge, 2012; Michaud, 2012; Neal et al., 2014). Plantar pressure analysis plays a crucial role in the evaluation, diagnosis, and characterization of gait patterns in patients (Zulkifli and Loh, 2020) and provides valuable insights into foot function. Widely used in clinical practice, it measures the distribution of pressure across different areas of the foot during various activities, such as standing, walking, and running (Ramirez-Bautista et al., 2018). Numerous studies have emphasized its importance, particularly in clinical settings, where plantar pressure data aid in assessing conditions such as diabetic foot ulcers (Fernando et al., 2016; Lockhart et al., 2024), foot misalignments as in flatfoot (Han et al., 2011; Khan et al., 2023), musculoskeletal disorders of the lower extremities, (Orlin and McPoil, 2000; Detels et al., 2024), and diseases of the central nervous centrum (Detels et al., 2024). Notably, pressure measuring systems have been developed to enhance ergonomic footwear design (Zulkifli and Loh, 2020).

Many tasks related to plantar pressure analysis rely heavily on the segmentation (also called zoning) of pressure profiles into specific areas of interest, such as the medial and lateral zones, to compare pressure distributions (e.g., pronation vs. supination). Comparative studies have demonstrated the diagnostic value of these segmented areas, particularly in identifying abnormal pressure patterns that may indicate underlying conditions (Periyasamy et al., 2011; Periyasamy and Anand, 2013; Cimolin et al., 2016). Segmentation approaches for plantar pressure data can be broadly categorized into two groups: (a) *proportion-based* and (b) *data-driven* approaches. Proportion-based methods (a) typically rely on predefined regions determined by foot length and width ratios. Various approaches can be found in the scientific literature (Cavanagh and Rodgers, 1987; Nyska et al., 1995; Deschamps et al., 2013; Wafai et al., 2015; Ramirez-Bautista et al., 2018). In the context of clinical assessment of the foot, the arch index proposed by Cavanagh and Rodgers (1987) is a widely used objective method for classifying foot type (high, normal, or flat arch), whereby the foot is divided into three parts, excluding the toes, to determine the ratio of the midfoot area to the area of the entire foot. Nyska et al. (1995) defined seven areas of interest (heel; midfoot; lateral, intermediate, and medial forefoot; toes 2–5; and the hallux), whereas Deschamps et al. (2013), Han et al. (2023) and Wafai et al. (2015) chose 10 areas of interest (medial and lateral heel, midfoot, each metatarsal, toes 2–5, hallux). Pauk et al. (2010) followed by classifying into five areas (toes, metatarsal heads (i.e., metatarsal areas), navicular bone, cuboid bone, and the heel). Ramirez-Bautista et al. (2018) proposed dividing the foot into 14 areas, marking every toe and metatarsal. These regions serve as templates and are scaled to fit the individual foot dimensions. Proportion-based approaches perform well when the data quality is high and the foot structure is relatively standard. However, in cases where the foot exhibits unique characteristics or the data quality is compromised, the segmentation results often require manual correction. This manual process is time-consuming, prone to subjective error, and becomes impractical when dealing with the large datasets generated by modern technologies, such as pressure plates integrated into treadmills.

As an alternative, machine learning (ML)-based approaches have emerged as potential solutions for plantar pressure segmentation. These methods excel in handling lower-quality data and nonstandard foot structures. Although several studies have demonstrated the potential of ML in this field, a significant research gap remains. To the best of our knowledge, only three studies have used deep learning techniques for automated foot segmentation into anatomically relevant zones (Wang et al., 2019; Wang et al., 2020; Han et al., 2021). A fully convolutional network was adopted by Wang et al. (2019) to extract vital regions of interest. Their model demonstrated superior performance compared to other algorithms, achieving a low error in regions of interest relative to expert ratings while also outperforming in terms of computational efficiency. However, the authors reported that they used data from 10 subjects with standardized measurements from only one laboratory. In addition, Wang et al. (2020) used a fully convolutional network to segment the plantar pressure images into anatomical structures, concluding that their research has high potential for future studies by showing good segmentation results using data from 60 subjects from a single laboratory. Finally, Han et al. (2021) used ML-segmentation to predict functional foot zones with high accuracy using a deep self-organizing map neural network. Collectively, these studies highlight both the high potential and critical need for continued research in this domain. In summary, the literature has shown that in general:

(i) Developing an ML segmentation model is possible. However, previous studies have thus far only focused on relatively few data samples from the same data source, laboratory, and measurement system although variations in hardware setups can lead to differences in the resolution of plantar pressure profiles. To the best of our knowledge, no analysis has been conducted on whether ML-based segmentation models for plantar pressure data are sufficiently robust to handle these variations, which is an important factor for multicenter data collection and application while also a critical requirement for making ML effective in biomechanics (Halilaj et al., 2018).
(ii) Although plantar pressure data have been used to distinguish between normal and abnormal hallux angles (Wu et al., 2020; Rozaqi et al., 2023), estimating the hallux angle using ML is underexplored. In this context, a significant gap in the literature exists on research in detecting the center of metatarsal area 1 although identifying this anatomical landmark can aid in diagnosing conditions such as hallux valgus by providing a more precise calculation of the hallux angle from the base. Mask-based segmentation is a common approach used in image-based ML research (Yu et al., 2023), and key point prediction techniques have also been applied in other contexts to identify specific coordinates (Khaki et al., 2020). In metatarsal area 1 center prediction, it is yet to be determined whether calculating from segmentation masks or directly predicting key coordinates such as estimating the hallux angle produces more accurate results.
(iii) Another issue lies in the calculation of the foot angle or the separation of the foot into lateral and medial zones based on anatomical landmarks rather than simple proportional divisions. Ardhianto et al. (2022) investigated the foot progression angle (FPA), defined as the angle formed between the direction of walking and the longitudinal axis of the foot, to indicate the orientation of the longitudinal axis of the foot during walking. Other studies, such as that by Chae et al. (2020), defined the foot rotation angle as the inner line connecting the outermost points of the foot. However, from an anatomical perspective, the longitudinal line through the foot, defined as the line between the heel base and second and third metatarsals, is commonly used and provides a more anatomically accurate representation of foot rotation (Ludwig, 2022). This approach avoids the influence of the first metatarsal and offers a more precise reflection of the anatomical axis. Consequently, this method can serve as a more anatomically justified separation line for defining the medial and lateral foot zones.

However, manual identification of landmarks in plantar pressure data by experts remains challenging, as toes are not always fully visible in plantar pressure data for drawing the described line. To the best of our knowledge, the potential of ML in this context has not yet been extensively explored. Because of the difficulty in visually pinpointing exact landmark locations, it is unclear whether predicting a shifted point along the line from the interdigital space to the heel center is more effective than directly predicting the interdigital space itself. This approach could leverage the better-defined characteristics of the pressure distribution because a shifted point may be more reliably associated with the data than less visible features, such as poorly defined toes or indistinct landmarks.

To address these research gaps, the current study aims to investigate the effectiveness of ML-based approaches for plantar pressure segmentation, specifically focusing on the following research questions:

(i) Can ML-based models achieve expert-level accuracy in foot segmentation into hallux, metatarsal area 1, metatarsal areas 2–5, and the heel based on multicenter data sources from different hardware configurations?
(ii) Does mask-based segmentation offer more accurate results in determining the center of metatarsal area 1 than key point prediction or an ensemble approach that calculates the mean of both methods?
(iii) Regarding the identification of landmarks for drawing an anatomical medial-lateral separation line, is predicting the shifted position of the interdigital space of the second and third toes more accurate than predicting a point directly within the interdigital space between the second and third toes?

## 2 Materials and methods

### 2.1 Workflow overview

**FIGURE 1:**
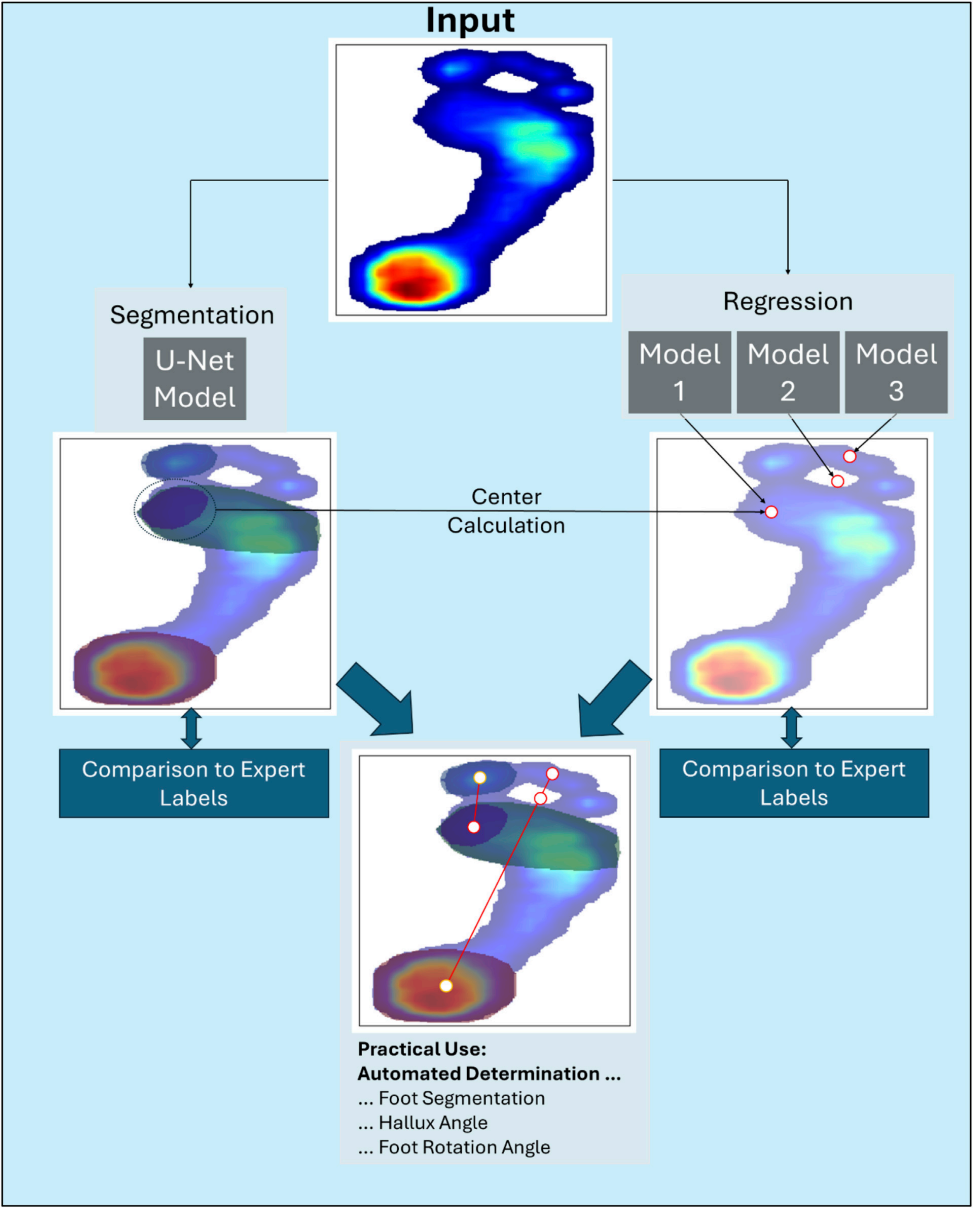
Workflow overview of the study. The segmentation areas and regression key points are listed and described in more detail in Table 1.

The workflow of this study is illustrated in Figure 1, which also provides a visual overview of the segmentation regions and predicted key points. Two distinct modeling approaches were used to address these research questions. The input data for all models consisted of preprocessed plantar pressure distribution maps. All models were trained in a supervised manner using plantar pressure data labeled by multiple experts (see Section 2.3).

The segmentation task employed a U-Net model to answer research question (i), by segmenting the plantar surface into four regions: the hallux, metatarsal area 1, metatarsal areas 2–5, and the heel. For the regression tasks that addressed research questions (ii) and (iii), three individually trained ML models were used (Models 1, 2, and 3). These models predicted the location of the interdigital space between toes 2 and 3 along with its shifted position and the center of metatarsal area 1 as the key points. Additionally, the center of metatarsal area 1, derived from the predicted segmentation of the U-Net model (illustrated by a horizontal arrow in Figure 1), was compared with the regression-based prediction of the same key point. Detailed descriptions of these regions and their key characteristics are provided in Table 1.

**TABLE 1:** Description of the segmentation areas and regression key points of the current prediction study. In case of no other references for the directly labeled regions, we referred to the regions shown in Bennetts et al. (2013), Han et al. (2023) and Wang et al. (2020).

Type of determination	Type	Target name	Description
Directly labeled	Segmentation Mask	Hallux	The hallux refers to the big toe, the first and largest toe
	Segmentation Mask	Heel	The heel is the rear part of the foot, primarily made up of the calcaneus bone
	Segmentation Mask	Metatarsal Areas 1–5	This region includes the metatarsals for the second through fifth toe, spanning across the midfoot. An isolated analysis of metatarsals (except metatarsal area 1) was not conducted because of the low separation accuracy between the individual metatarsals from plantar pressure images
	Segmentation Mask	Metatarsal Area 1	This area encompasses the first metatarsal bone, which is located at the base of the hallux
	Coordinates	Interdigital Space 2–3	This refers to the area between the second and third toes, where soft tissue and small spaces exist between the metatarsal bones. The middle of the second toe was used as the height reference to overcome differences in foot forms (Waldt and Wörtler, 2014)
Determined based on labeling	Coordinates	Key Point Center Metatarsal Area 1	This point is calculated using the center of the labeled hallux segment. The center is determined by computing the median of the x- and y-coordinates of the pixels within the hallux region to ensure robust center estimation, even in the presence of irregular shapes
	Coordinates	Key Point Shifted Interdigital Space 2–3	The coordinates for this point are derived by using the original labeled point of the interdigital space 2–3 and the labeled heel segment (Waldt and Wörtler, 2014). First, the center of the heel segment is calculated similar to the center of metatarsal area 1. A line is then drawn from the interdigital space 2–3 to the heel center. The point along this line that corresponds to the highest possible location within the metatarsal area (covering metatarsals 1–5) is selected as the shifted coordinate

All predictions were evaluated against expert-labeled data, which served as the ground truth for this study.

### 2.2 Subjects and data

Only individuals of legal age were eligible to participate. The participants were informed of the study procedures and applicable data protection regulations, after which they provided informed consent. The study adhered to the principles outlined in the Declaration of Helsinki and was approved by the institutional ethics committees. Data collection was performed at multiple centers using two different measurement systems: RSscan resistive pressure sensor plates (RSscan Lab Ltd., Ipswich, England) and fused deposition modeling (FDM) capacitive pressure sensor plate (Zebris Medical GmbH, Isny, Germany). The plantar pressure data for the left and right feet of each participant were recorded. The measurements were conducted barefoot with instructions to ensure compliance. Recordings were conducted under both static (53%) and dynamic conditions (47%). For static trials, participants stood barefoot in an upright position on the pressure platform for 10 s (sampling rate: 50 Hz). After a 60 s habituation period on a treadmill, dynamic trials comprised three overground walking passes at each participant’s self-selected speed (sampling rate: 100 Hz). For the dynamic recordings, a summarized plantar pressure profile was created using data from a valid stride and peak pressure values, as commonly reported in the literature (Arzehgar et al., 2025). All plantar pressure profiles were aligned along the long axis, with toes oriented at the top and heel at the bottom of the image. Duplicate or erroneous entries were identified and removed prior to analysis. Outliers caused by factors such as wearing shoes or measurement errors were manually reviewed by an expert who determined their inclusion or exclusion. This resulted in a final dataset of 758 plantar pressure foot samples from 460 participants (197 females, 263 males). We limited data collection to essential variables to align with the study’s focus on model development and evaluation rather than biomechanical subject characterization or exploration. Therefore, additional anthropometric or descriptive participant data were not collected, to minimize data and ensure participant privacy. This approach ensured compliance with the principle of data minimization [GDPR Art. 5 (1) (c)] (European Union, 2016), which stipulates that personal data must be “adequate, relevant, and limited to what is necessary” for the intended processing purposes.

### 2.3 Data labeling

Raw plantar pressure data, originally available at various resolutions from different hardware setups, were standardized for visual labeling through a series of preprocessing steps. First, the data were uniformly upsampled via bilinear interpolation to ensure a consistent base resolution. To preserve the original proportions of the foot pressure distribution, each pressure map was resized while maintaining its original aspect ratio. This was achieved by scaling both height and width proportionally to fit within a target resolution of 300 × 100 pixels. Following this aspect ratio–preserving upsampling, the resized maps were symmetrically padded with zero values to reach a final standardized size of 310 × 110 pixels. The additional padding was intentionally added to provide a narrow margin of empty space around the pressure areas, which facilitated more comfortable and precise annotation in the labeling software. The choice of the resolution was informed by a preliminary evaluation in the labeling software tool, where three clinical experts—each with multiple years of experience in plantar pressure analysis—were presented with several upsampled pixel sizes. All three experts independently selected the selected resolution as the most suitable for accurate and consistent data labeling.

To enhance the visual interpretability of the pressure maps, a smoothing operation was implicitly incorporated during upsampling to reduce artifacts and improve clarity. Additionally, a custom color map was applied to the processed data. This colormap was derived from matplotlib’s “jet” colormap (Hunter, 2007) and modified to display minimum values (zero pressure regions) in white, to enhance the contrast between regions of no activity and areas of varying pressure intensity. This representation aligns with the established practices in plantar pressure analysis (Kirtley, 2006) and facilitates interpretation for domain experts familiar with similar visualizations.

The processed data were then exported as high-resolution images in portable network graphics (PNG) format. Visualizations excluded axes and color bars to reduce distractions, emphasizing the spatial patterns in pressure data. These standardized, high-quality visualizations were then randomly assigned to three experts, who performed the labeling using the open-source Python tool *Labelme* (Wada, 2018). During this process, the labels described in Section 2.1 were identified. The labels were then reviewed by another expert, with particular attention paid to labels that potentially contained human errors in placement. These samples were re-evaluated and necessary adjustments were made through a collaborative dialogue involving at least two experts. After labeling, the center of metatarsal area 1 and shifted interdigital space 2–3 were determined based on the procedure described in Section 2.1.

To address potential uncertainties arising from human labeling variability, an exploratory analysis of inter-rater reliability was performed on a randomly selected subset of 30 plantar pressure samples. As described in Section 2.7, three independent raters—each with several years of experience in plantar pressure analysis—manually annotated the subset. For comparability, the manually annotated data were subsequently resized to match the input shape used in the modeling pipeline (see Section 2.4). Inter-rater reliability was quantified using the same evaluation metrics (Intersection over Union, Dice coefficient, Euclidean distance) employed for assessing model performance (see Section 2.7). For each image, pairwise comparisons between raters were aggregated by calculating the mean.

This methodological choice was motivated by the limitations of traditional agreement metrics such as Fleiss’ Kappa, which are primarily designed for categorical data and are known to be sensitive to class imbalance. In image segmentation tasks, especially those involving masks with large background areas, such sensitivity can distort the assessment of agreement. In contrast, overlap-based metrics are more appropriate for evaluating segmentation reliability, as they are less affected by the predominance of background pixels and better reflect the spatial congruence of annotated regions (Taha and Hanbury, 2015). Moreover, applying the same evaluation metrics to both human annotations and model outputs facilitates direct comparison, providing a more interpretable benchmark of model performance relative to expert human raters.

### 2.4 Data preprocessing

To train the ML models, the raw plantar pressure maps (note that this refers to the unprocessed data, not the preprocessed data used for labeling) were normalized using min-max scaling to a range between [0, 1]. This normalization preserved the relative intensity variations in the pressure data while ensuring uniform scaling across samples. The normalized pressure maps were then resized to 256 × 256 pixels using bilinear interpolation for the images and nearest-neighbor interpolation for the segmentation masks created via labeling. To determine whether 256 × 256, which is commonly use as input size for U-Nets (Nazeri et al., 2018; Li, 2024), we compared it to two alternative resolutions: 128 × 128 and 512 × 512. Training at 128 × 128 reduced epoch time by approximately 30%, but resulted in a 10% drop in validation set segmentation performance, based on the median Dice score (see Section 2.7). In contrast, using 512 × 512 increased computational cost by over 100%, while still causing a 4% decrease in the median Dice score. Therefore, 256 × 256 was selected for all subsequent experiments. The coordinates of the key points obtained during labeling were further rescaled to the resized pressure maps. Both the pressure maps and segmentation masks were preprocessed using OpenCV (Bradski G., 2000).

Segmentation masks were processed using one-hot encoding, and categorical representations were converted into a binary format along separate channels. This resulted in a final target feature set with dimensions of n × 256 × 256 × 4, where n is the number of samples, 256 × 256 is the spatial resolution, and the four channels correspond to the segmented areas of interest.

### 2.5 Segmentation model

Among segmentation models, the U-Net architecture has emerged as one of the most widely used and effective approaches. The U-Net model is a convolutional neural network designed specifically for image segmentation. It comprises two main parts: contracting (downsampling) and expansive (upsampling). The contracting path captures context by applying successive convolutional layers and pooling operations to reduce the spatial dimensions while increasing the number of feature channels, thereby effectively extracting high-level features from the input image. The expansive path reconstructs the output segmentation map by upsampling the feature maps and concatenating them with the corresponding feature maps from the contracting path. This skip connection helps retain the spatial information lost during downsampling. The final output is a pixel-wise classification map that allows the precise delineation of target objects within the input image (Ronneberger et al., 2015).

Studies have demonstrated the effectiveness of the U-Net model in outperforming traditional segmentation methods in various medical fields, including tumor detection, organ segmentation, and orthopedic assessments (Jain et al., 2021; Ghulam et al., 2023; Kasliwal et al., 2024; Tiribilli and Bocchi, 2024). The U-Net architecture is particularly effective for tasks with limited training data because of its ability to simultaneously learn from context and spatial information (Ronneberger et al., 2015; Azad et al., 2022). U-Net models have been successfully applied in the domain of ML-based plantar pressure segmentation (Bai et al., 2021). While alternative architectures such as Mask R-CNN (He et al., 2017) and Vision Transformers adapted for segmentation (e.g., Hatamizadeh et al., 2022) have shown promise, their reliance on large-scale datasets for optimal performance (Al-Hammuri et al., 2023) renders them less suitable for this study. In contrast, the U-Net architecture excels in scenarios with limited data, leveraging its unique design to preserve intricate anatomical features (Isensee et al., 2021). Considering these advantages, the U-Net architecture was selected as the foundation for this study.

Data augmentation was applied during the training of the U-Net model to increase data diversity, help the model learn more robust features, and improve its accuracy on unseen data. The transformations simulate real-world variations to improve the generalizability of the model. The augmentation pipeline used the Keras ImageDataGenerator package (Chollet, 2015), applying the following transformations to both the images and corresponding masks:

• Rotation: Random rotations within a range of 20°.
• Zoom: Random zoom within a range of 10%.
• Shift: Random width and height shifts within a range of 10%.
• Flip: Horizontal flipping of the images and masks.
• Shear: Random shear within a range of 5°.
• Fill mode: Set to ‘nearest’ to avoid zero-filling when transforming the images and masks.

**FIGURE 2:**
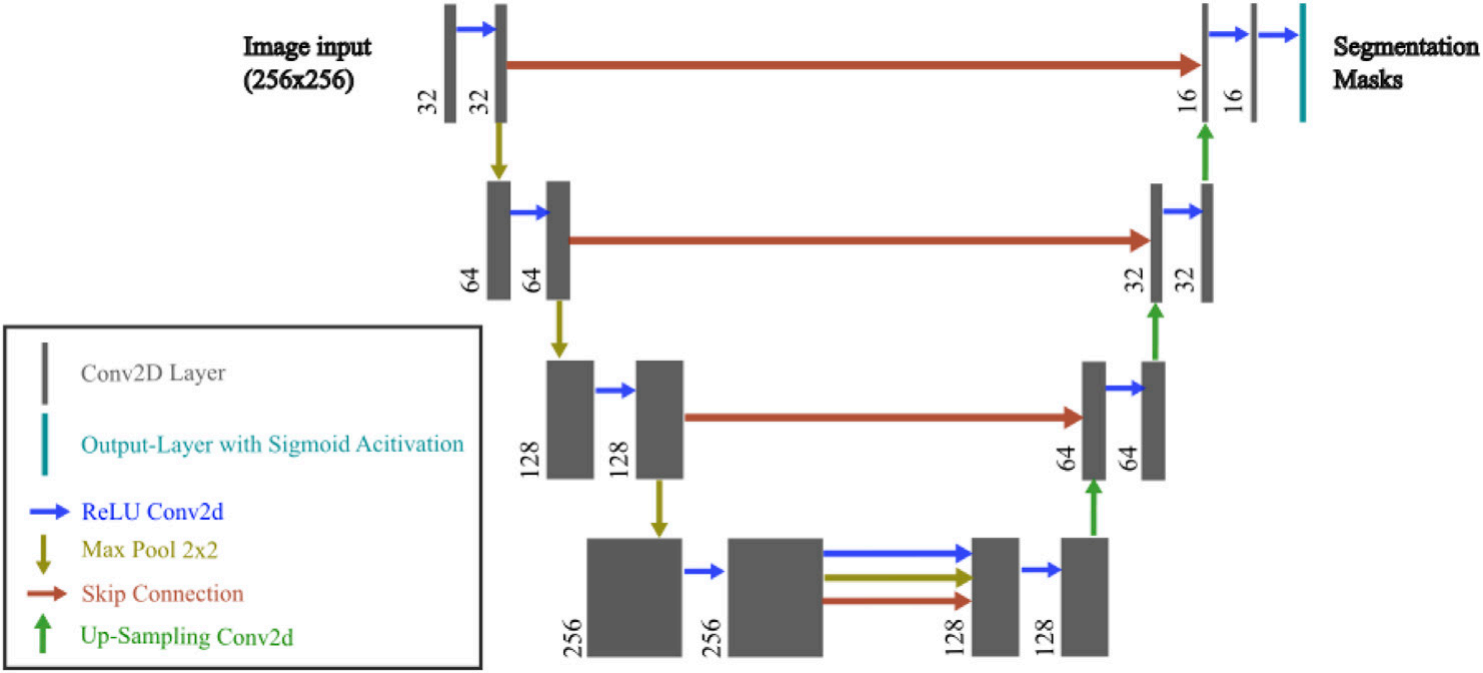
U-Net model used in the current study. The architecture consists of an encoder and decoder with skip connections. Each convolutional layer uses a 3 × 3 kernel, followed by batch normalization and LeakyReLU activation. The encoder progressively downsamples the input image through Conv2D and MaxPooling2D layers, while the decoder upsamples the feature maps using UpSampling2D and concatenates the corresponding encoder layers. Dropout is applied in the bottleneck layer to prevent overfitting. The final output layer has four channels with a sigmoid activation function.

The model architecture and hyperparameter tuning were manually optimized based on validation Mean Intersection over Union (MIoU) (threshold = 0.5; see Section 2.7) across the target masks. Hyperparameter search was done with 3 × 3 kernels with various filter depths (16–256), and three to five encoder–decoder stages. Dropout rates ranging from 0.3 to 0.6 were evaluated at various positions, including the option of applying no dropout. We also compared different loss functions (e.g., binary cross-entropy vs. Dice loss) and initial learning rates (1e-2 to 1e-5). Parameter ranges were informed by established biomedical U-Net designs (Ronneberger et al., 2015; Isensee et al., 2021; Azad et al., 2022; Ghulam et al., 2023). Manual tuning was preferred over automated searches, as domain expertise allowed for targeted exploration of promising configurations and early exclusion of suboptimal ones. The resulting model configuration is shown in Figure 2. The final model was compiled using the adaptive moment estimation (Adam) optimizer with an initial learning rate of 0.0001 and a binary cross-entropy loss function as the segmentation task involving multiple classes with overlapping regions (between metatarsal area 1 and metatarsal areas 1–5).

Training was conducted in epochs, each consisting of 150 steps, with plantar pressure data and corresponding masks loaded in batches of size 16. To enhance the training efficiency, an adaptive learning rate strategy was implemented such that if the validation performance did not improve for five consecutive epochs, the learning rate was reduced by a factor of 0.5. Early stopping was also employed to mitigate overfitting and halt training if the validation metric failed to improve after five epochs. During the training, the segmentation quality was assessed using MIoU across the four classes (threshold = 0.5; see Section 2.7).

### 2.6 Regression models

The effectiveness of regression-based approaches for tasks involving spatial predictions such as coordinate points has been previously presented (Jiang, 2019). In this study, a hybrid model that combines a pretrained U-Net for segmentation with a regression head for 2D spatial coordinate prediction is proposed. By utilizing the latent features from the pretrained U-Net model, this architecture integrates U-Net’s robust feature extraction capabilities with a lightweight, trainable regression head specifically optimized for accurate coordinate prediction.

The U-Net was loaded with frozen weights to retain its feature extraction capability without further updates during training. For the regression model, a manual hyperparameter search (e.g., variations in learning rates, dropout rates, batch sizes, number of dense units, and presence or absence of residual connections)—similar to the procedure used for the U-Net model—was conducted based on the average validation Euclidean distance of the predicted coordinate points across the three tasks. The details of the regression head are as follows:

• Latent Feature Processing:
o Conv2D Layer 1: 3 × 33, 128 filters, ReLU activation, same padding
o Batch Normalization Layer: Applied after Conv2D Layer 1
o Dropout Layer: Dropout probability = 0.2
o Conv2D Layer 2: 3 × 33, 64 filters, ReLU activation, same padding
o Batch Normalization Layer: Applied after Conv2D Layer 2
• Dimensionality Reduction:
o Global Average Pooling Layer: Reduces spatial dimensions into a compact feature vector
• Feature Refinement with Residual Block:
o Dense Layer 1: 128 units, ReLU activation
o Batch Normalization Layer: Applied after Dense Layer 1
• Residual Block:
o Dense Layer 2: 128 units, ReLU activation
o Batch Normalization Layer: Applied after Dense Layer 2
o Dense Layer 3: 128 units
o Add Layer (Shortcut): Combines input to the residual block with an output of Dense Layer 3
• Output Layer:
o Dense Layer: Two units (linear activation) predict the x- and y-coordinates.

The model was compiled using the Adam optimizer (initial learning rate: 0.001) and Huber loss. The Huber loss was selected because it is robust to outliers (Terven et al., 2023) arising from human errors during labeling. Such errors are expected to some extent, given the challenges that human experts face in accurately and visually identifying key points of interest. The training was enhanced using callbacks, including learning rate adjustment (reducing learning rate by half after five epochs without improvement), model checkpointing (the best model saved based on validation performance), and early stopping (training stopped after eight epochs of no improvement, restoring best weights). The Euclidean distance was computed as a validation metric.

To ensure a fair performance comparison and attribute the differences in model performance to the task itself, model hyperparameters were standardized across the three models to predict the key points of the center metatarsal area 1, interdigital space 2–3, and adjusted interdigital space 2–3. Notably, while the hyperparameters remained consistent, a separate model was trained for each target.

### 2.7 Evaluation

To ensure a robust model evaluation, a grouped k-fold cross-validation strategy was employed with five splits. This method accounts for participant-based grouping, where each participant contributes multiple data samples (e.g., dynamic and static conditions, as well as left- and right-foot data). The grouped k-fold ensured that the data included were not from a single participant in either the training or test sets, minimizing the risk of overfitting to participant-specific characteristics. For each fold, the dataset was partitioned into approximately 70% training, 10% validation, and 20% testing data, preserving group information across the splits.

For the segmentation task, the Intersection over Union (IoU) and Dice coefficient (Dice score) were calculated for each segmented area after applying a threshold of 0.5 to minimize unintended mask areas in the U-Net outputs. The IoU measures the overlap between the predicted and ground-truth segmentation masks and is calculated by dividing the intersection of the predicted and true regions by their union. The IoU ranges from 0 to 1, where 0 indicates no overlap and 1 indicates perfect overlap. This metric is particularly useful for evaluating segmentation performance when dealing with imbalanced classes (Rahman and Wang, 2016). The Dice coefficient is another metric that is used to evaluate the overlap between two binary sets. Similar to the IoU, it is generally more sensitive to small differences between the predicted and ground-truth regions. The Dice score ranges from 0 to 1, where 1 represents perfect agreement and 0 indicates no overlap (Bertels et al., 2019).

To predict the x and y coordinates of key points, the Euclidean distance was computed. Because the images have a consistent scale, the Euclidean distance provides a meaningful and reliable measure of prediction accuracy without the need for normalization. Evaluation metrics are reported as the median with median absolute deviation (MAD) across the cross-validation folds as the median and MAD are less influenced by outliers and skewed data distributions. Additionally, a bootstrap 95% confidence interval (CI) of the median is calculated based on 1,000 samples.

## 3 Results

### 3.1 General foot segmentation results

The results of the U-Net model evaluation of the cross-validation procedure indicate strong alignment between the expert-based and U-Net-generated segmentations (Table 2). The highest overlap, as measured by both IoU and Dice score, is observed in the Heel segment, followed by the Metatarsal areas 1-5 and the Hallux. The lowest overlap is noted in Metatarsal area 1.

**TABLE 2:** Test set performances separate for each targeted segmentation area during 5-fold cross-validation. MAD (median absolute deviation) is reported as a robust measure of variability. Values are reported with corresponding 95% bootstrap confidence intervals based on 1,000 resamples.

Segment	Dice score		Intersection over union	
	Median [95% CI]	MAD	Median [95% CI]	MAD
Heel	0.96 [0.92, 0.99]	0.01	0.92 [0.90, 0.94]	0.02
Metatarsal Areas 1–5	0.95 [0.91, 0.98]	0.01	0.91 [0.89, 0.92]	0.02
Hallux	0.92 [0.89, 0.96]	0.03	0.85 [0.80, 0.88]	0.05
Metatarsal Area 1	0.88 [0.83, 0.90]	0.04	0.78 [0.72, 0.82]	0.06

**FIGURE 3:**
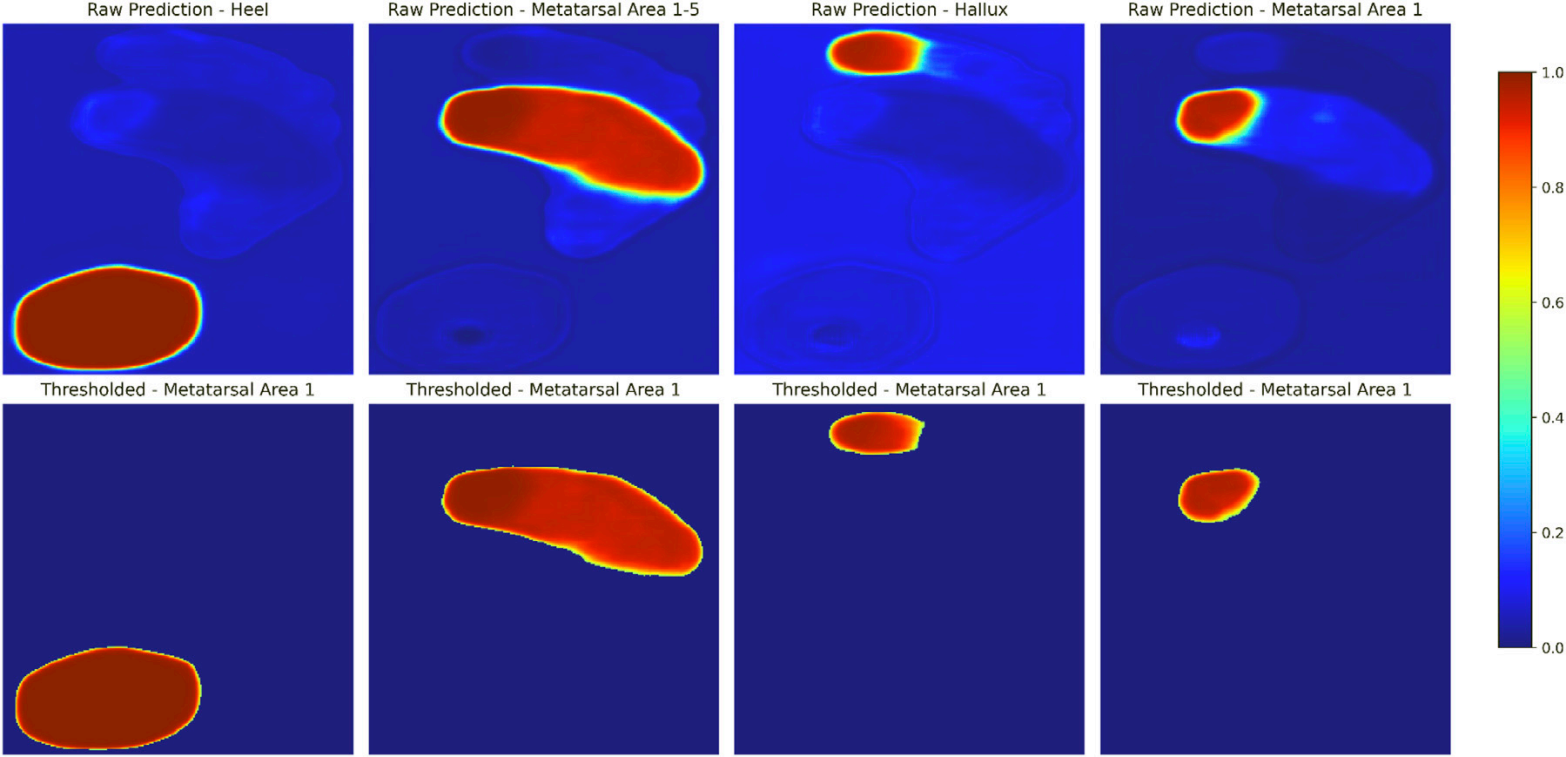
Example of U-Net-based segmentation for a single participant’s foot. Shown are model-generated predictions for each output channel, representing different segmentation areas, are shown before (upper panels) and after (lower panels) applying a threshold of 0.5. The colorbar represents the predicted probability values of the mask, ranging from 0 to 1, with higher values indicating stronger confidence in the predicted segmentation. Note that only model-based annotations are shown in this figure. For a visual comparison of expert annotations and thresholded model predictions overlaid on normalized and rescaled plantar pressure profiles, refer to Figure 4 (upper example).

**FIGURE 4:**
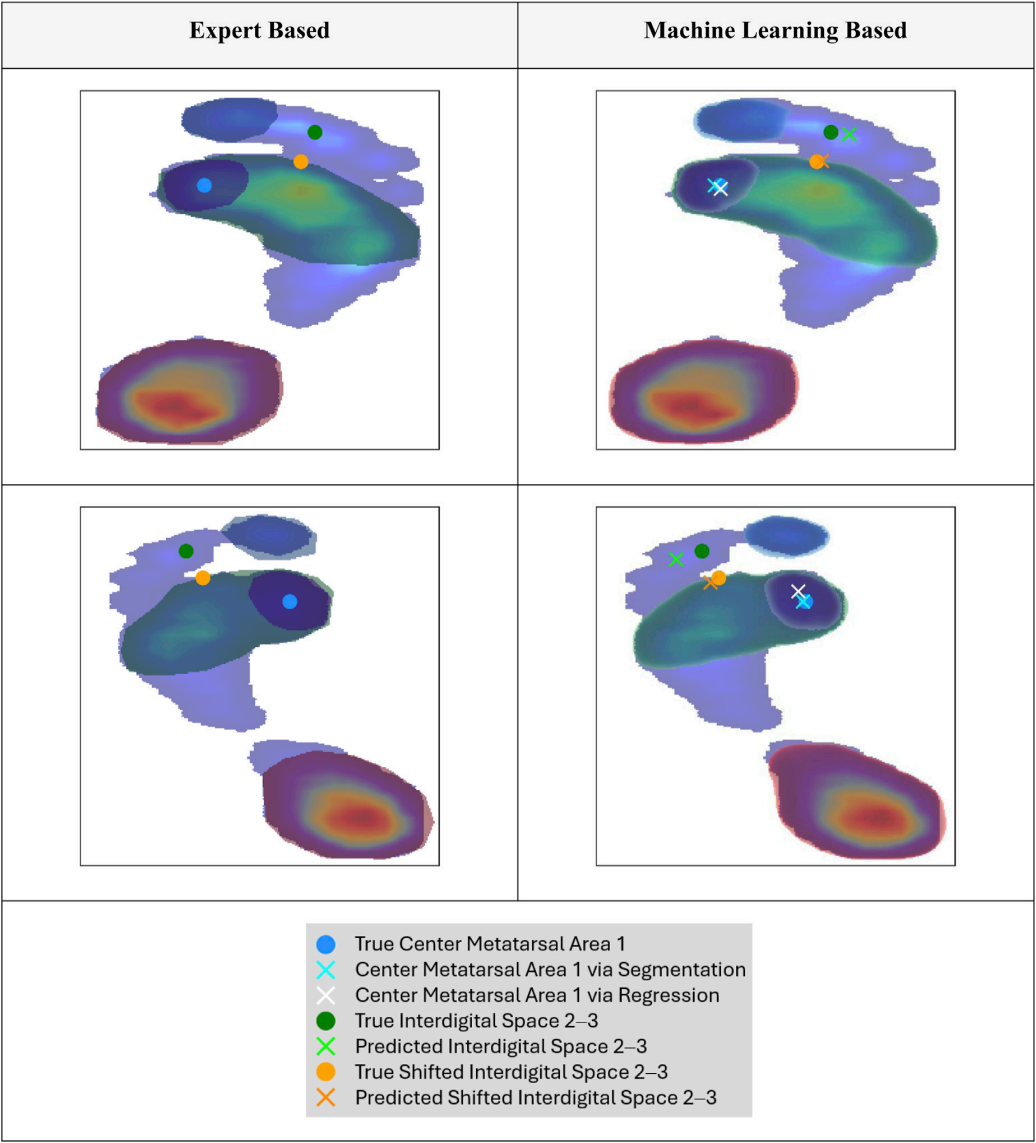
Exemplary normalized and rescaled plantar pressure profile with overlaid segmentation masks: expert annotation (left) and model-predicted, thresholded mask (right; see Figure 3 for thresholding details). True and predicted key points generated using different approaches are also shown (see Section 3.2 for methodological details).

The U-Net-based segmentation for a single participant’s foot shown in Figure 3 includes thresholding. The results demonstrate that applying a threshold of 0.5 minimizes unintended mask areas in the U-Net outputs. Additional examples are shown in Figure 4, where excellent alignment between expert-based and predicted segmentation is observed.

### 3.2 Key point prediction task

The results of the key point-prediction tasks are presented in Table 3. Using the segmentation mask of metatarsal area 1 generated by the developed U-Net models yielded higher spatial accuracy than directly predicting the coordinates with the regression model or ensemble predictions.

**TABLE 3:** Key point prediction results, including the median and MAD (median absolute deviation; robust measure of variability), of the Euclidean distance between the true and predicted coordinates. Values are reported with corresponding 95% bootstrap confidence intervals based on 1,000 resamples.

Center/Key point	Median [95% CI]	MAD
Center Metatarsal Area 1 via Segmentation	4.47 [4.08, 4.75]	2.24
Center Metatarsal Area 1 via Regression	7.72 [7.24, 8.10]	3.34
Ensemble	5.26 [4.98, 5.61]	2.27
Interdigital Space 2–3	10.06 [9.38, 10.60]	4.60
Shifted Interdigital Space 2–3	8.34 [7.88, 8.90]	3.85

**FIGURE 5:**
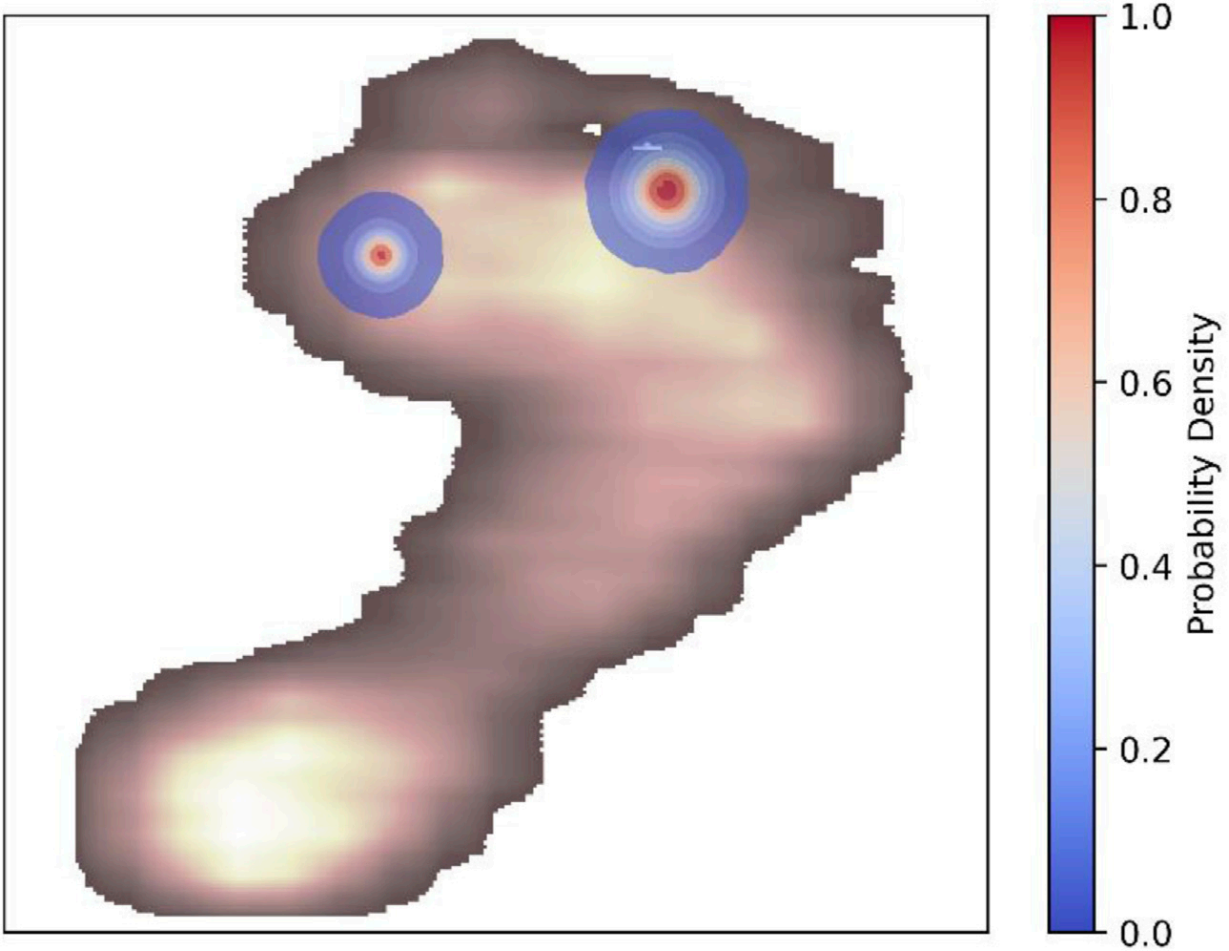
Euclidean distance differences between predicted and actual points across all samples are used to estimate the Gaussian kernel density for the center of metatarsal area 1 via segmentation and shifted interdigital space 2–3. The kernel density contours represent the probability density of these errors. An example of plantar pressure distribution is shown to facilitate error comparison relative to foot size. A customized colormap for the plantar pressure distribution was used to improve visibility of the kernel density contours.

Regarding the prediction of a reference point for anatomical foot separation into lateral and medial sections, shifting the key point location of the interdigital space 2–3 led to an improvement in spatial accuracy compared to directly predicting the coordinates of the interdigital space 2–3. Therefore, the spatial accuracy of the shifted interdigital space prediction was lower than that of the metatarsal area 1 center prediction. Example predictions of the key points are compared with the ground truth in Figure 4. When focusing on the best-performing approaches (center metatarsal area 1 via segmentation and shifted interdigital space 2–3), both the median spatial error and error deviation were higher for the shifted interdigital space 2–3. The distribution of errors derived from all test samples is shown in Figure 5 to facilitate a visual comparison of the errors in relation to foot size.

### 3.3 Exploratory analysis of inter-rater reliability

**FIGURE 6:**
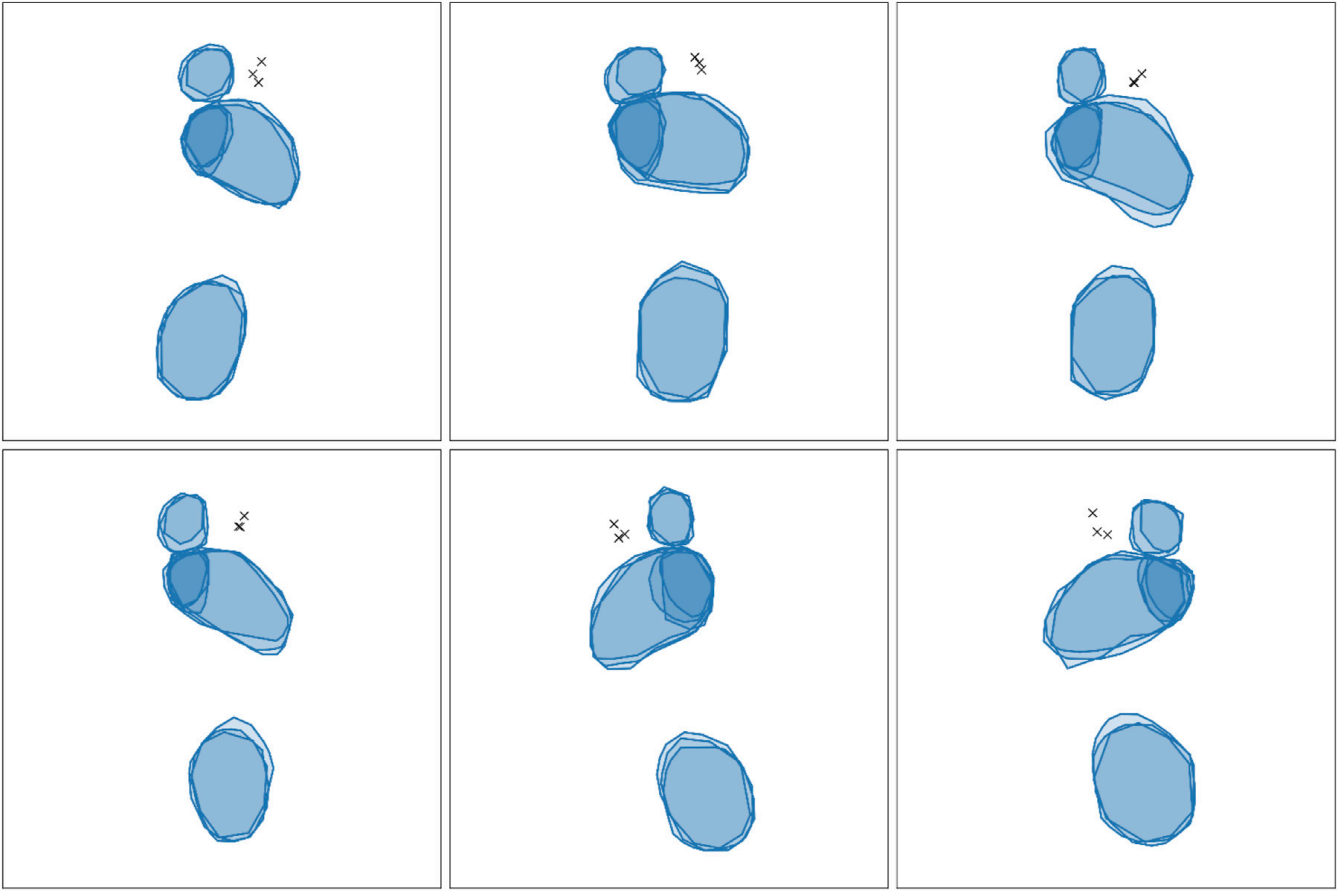
Exemplary inter-rater reliability of three raters annotating the anatomical landmarks. Semi-transparent filled polygons represent individual annotations, with overlapping regions visualized through increased color density. Solid borders highlight individual outlines. Note: The annotations shown reflect the original shapes as provided by the raters prior to resizing to the model’s input shape, preserving the spatial characteristics of the raw labeling process.

Figure 6 illustrates examples of spatial agreement among the three human raters. Quantitative metrics describing overlap for each anatomic segment are summarized in Table 4. For the interdigital space 2–3, the Euclidean distance between corresponding landmark coordinates yielded a median of 13.69 (95% CI: 12.51–17.37) with a MAD of 3.63.

**TABLE 4:** Inter-rater reliability metrics for three experts across anatomical segmentation regions. Median [95% CI] and MAD are shown for Dice score and Intersection over Union, with 95% confidence intervals obtained via 1,000 bootstrap resamples. (MAD = median absolute deviation). Metrics are computed on annotations resized to match the model’s input shape, ensuring comparability with the evaluation of model performance.

Segment	Dice score		Intersection over union	
	Median [95% CI]	MAD	Median [95% CI]	MAD
Heel	0.93 [0.92, 0.94]	0.01	0.87 [0.86, 0.88]	0.01
Metatarsal Areas 1–5	0.92 [0.92, 0.93]	0.01	0.86 [0.85, 0.86]	0.01
Hallux	0.86 [0.84, 0.88]	0.03	0.75 [0.73, 0.77]	0.03
Metatarsal Area 1	0.85 [0.84, 0.86]	0.02	0.72 [0.71, 0.74]	0.01

## 4 Discussion

The segmentation models demonstrated high alignment between expert-based and U-Net-generated segmentation. Among the segmented areas, the overlap between expert and ML-based segmentation areas was the highest for the heel, followed by metatarsal areas 1–5, the hallux, and metatarsal area 1. These findings indicate that ML-based models, particularly U-Net architectures, can achieve near-expert-level accuracy in foot segmentation, particularly in the heel, metatarsal areas 1–5, and hallux.

The overlap between the predicted and expert-labeled areas was the lowest for metatarsal area 1. A likely explanation is that U-Net models excel at segmenting areas with clear, visually separable borders but perform less effectively in regions defined primarily by relative positions without distinct boundaries (Kumar et al., 2024; Xu et al., 2025). Metatarsal area 1, which is less distinct in terms of plantar pressure, exemplifies this limitation. The experts noted that metatarsal area 1 was the most difficult to segment, suggesting that this area may exhibit inherently higher variability and subjectivity—both for human annotators and ML models. The level of accuracy required from such models is highly dependent on the intended application: for exploratory, data-driven research, the current segmentation performance may be sufficient and justifiable—especially when the goal is to efficiently analyze large datasets. In contrast, clinical scenarios such as diagnostics or surgical planning demand more precise and interpretable outputs. In these cases, segmentation models should be considered as automated preprocessing tools that assist, but do not replace, clinical expertise (“doctor-in-the-loop” approach (Kieseberg et al., 2015; Kieseberg et al., 2016)). This distinction becomes particularly relevant for metatarsal area 1, where we observed comparatively higher segmentation errors.

The model demonstrated robust performance across various measurement devices despite having been trained on data from different pressure plates. Preprocessing steps, such as size normalization and scaling of raw plantar pressure profiles, possibly contributed to reducing discrepancies between data sources, thus enabling the model to effectively handle data from diverse hardware setups. Additionally, the relatively low variance observed during cross-validation indicates consistent results and robustness to variations in the training data. Nonetheless, despite encouraging cross-validation performance, the model’s generalizability to completely unseen hardware configurations has not been empirically tested, and prior work shows that domain shift across imaging devices can substantially impact segmentation accuracy (Yan et al., 2019). Future work should include validation on additional hardware from new centers and investigate domain adaptation strategies, such as unsupervised domain adaptation (Perone et al., 2019), to further enhance robustness across device-specific variability.

The segmentation results revealed that metatarsal area 1 was the most challenging region for segmentation. This may be linked to the anatomical structures of different feet, especially the foot arch (Xiong et al., 2010), which leads to higher pressure on the metatarsal heads (Periyasamy and Anand, 2013) and therefore to a mis-segmentation due to shifted peak values aside from metatarsal area 1. Notably, using a regression model to detect the center of metatarsal area 1 did not directly improve the calculations using the segmentation model. Furthermore, an ensemble approach that combined coordinates from both regression- and segmentation-based methods via mean calculation did not enhance the accuracy of center detection. Consequently, this study advocates the use of a U-Net segmentation model over coordinate prediction using deep regression models, to identify the center of the first metatarsal area. When determining the hallux angle and considering the spatial error distribution in relation to the actual foot size (Figure 5), modeling results must be interpreted within the context of their application. For instance, in clinical applications where precise measurements of the foot structure are required, even small errors can have a significant impact. By contrast, for gait analysis in sports science, such deviations may be less consequential. Potential errors stemming from the labeling process, which can affect both model training and evaluation, must be carefully considered and are discussed further in this section.

The results revealed higher errors for key point detection of the interdigital space 2–3 compared to detecting the center of metatarsal area 1. Notably, shifting the key point location of interdigital space 2–3 to a potentially more detectable anatomical position led to a small improvement in spatial accuracy. This outcome suggests that the hypothesized improvement expected from shifting the key points is supported by the present study although the observed effect size was small. Summarizing these findings, the results indicate that ML-based detection of the interdigital space 2–3 has the potential to enhance the anatomical separation of the foot into medial and lateral zones, thereby facilitating the determination of the overall foot angle. However, the accuracy achieved in this study still leaves room for improvement because the observed errors highlight the need for manual evaluation and adjustment by experts. Consequently, the model, in its current state, should primarily serve as a preparatory step for identifying key points from a practical perspective. Several strategies may improve the accuracy: Exploring other shifted locations for the interdigital key point spaces 2–3 can help identify the anatomical separation line. Additionally, given that a higher accuracy was achieved for the coordinate detection of metatarsal area 1 center using mask-based segmentation, defining a segment for a representative area, such as toes 2 and 3 may be worthwhile in deriving a key point from the predicted segment. Furthermore, several optimization opportunities remain to be explored. For instance, tailoring model configurations to each specific regression task—rather than using a shared configuration for comparability—could improve task-specific performance. Additionally, applying augmentation techniques or experimenting with different input representations can help more effective model generalization to challenging key point locations. Another promising direction is the unification of segmentation and regression tasks within a multi-task learning architecture, which could enhance both performance and training efficiency.

Several limitations and key aspects must be considered when interpreting the results. The annotation process faced significant challenges, particularly in labeling the metatarsal area 1 and interdigital space 2–3. Experts reported difficulties in these regions arising from a lack of distinct key points, which hindered precise labeling. This aligns with the findings in the literature, acknowledging such ambiguities as potential sources of error (Guldemond et al., 2006). These challenges may also be reflected in model performance, indicating that the observed errors can be attributed to the inherent difficulty in interpreting plantar pressure profiles in these areas or potential labeling errors in human-generated labels, which can adversely impact model training.

When comparing inter-rater agreement to the model’s performance against the expert-defined ground truth, two key observations emerge. First, the three human raters exhibit slightly lower overlap scores across segmented regions than the model. Second, their point-to-point variability, particularly in the interdigital space 2–3, exceeds that of the model relative to the reference annotations. This discrepancy likely stems from the structured approach used to generate the ground truth: (1) independent annotations by three experts, (2) peer review by a fourth expert, and (3) collaborative resolution of discrepancies. That the model’s predictions align more closely with this refined consensus than the individual expert annotations do with each other underscores two important points: (a) residual variability persists in manual annotation, even among trained experts, and (b) the model successfully learns to replicate the collaboratively validated standard. Thus, the model achieves—if not surpasses—human-level agreement when evaluated against a consensus-based benchmark.

Underlying these observations are two primary sources of uncertainty categorized into aleatoric uncertainty, which stems from the inherent noise or variability in the data (e.g., measurement inaccuracies, sensor limitations, or inherent biological variability), and epistemic uncertainty, which arises from the lack of knowledge or gaps in model understanding, such as limited training data, small labeling errors, or incomplete model architecture (Shaker and Hüllermeier, 2020). Future research should investigate the validity and reliability of human labeling in this context, including systematic analysis of how the visualization and interface design employed during labeling may influence rater consistency, to better understand its impact on model training and evaluation. Such studies can help clarify errors stemming from both data labeling and model deficiencies, thereby informing strategies to optimize both annotation protocols and model development.

This study relied on manual hyperparameter tuning, which may not have exhaustively explored all potential parameter combinations. For a fair comparison, model hyperparameters were standardized across tasks. However, this approach may limit task-specific optimization. Therefore, automated hyperparameter optimization methods (Yu and Zhu, 2020) should be considered in future research to identify the most effective parameter configurations for each task and reduce potential biases. Finally, separate models were used for the segmentation and regression tasks. Although this approach facilitates independent performance evaluations, it may not fully exploit the synergy between these tasks. Future studies should explore integrating segmentation and regression into a single, unified model. Such an approach can enhance computational efficiency and improve overall prediction accuracy by leveraging shared features across tasks. Furthermore, should the dataset size increase in future work, it would be valuable to evaluate and compare the inclusion of additional input features (e.g., temporal plantar pressure data), incorporation of physics-informed modeling approaches (e.g., embed biomechanical knowledge, including foot-ground contact models) and the performance of alternative architectures—such as Mask R-CNN (He et al., 2017) and Transformer-based models (Hatamizadeh et al., 2022)—against the U-Net. These architectures, while currently constrained by their dependence on large-scale training data (Al-Hammuri et al., 2023), have demonstrated strong potential in segmentation tasks and may outperform U-Net under data-rich conditions. Finally, the segmentation and regression models employed in this study function as black box systems because of the lack of transparency in the decision-making processes and specific plantar pressure regions prioritized for predictions. This lack of transparency complicates error analysis, undermines trust in model outputs, and conflicts with General Data Protection Regulation (GDPR) compliance (European Union, 2016). To address this issue, recent works have highlighted the growing importance of explainable artificial intelligence (XAI) techniques in biomechanics (Dindorf et al., 2025). Methods like Grad-CAM (Selvaraju et al., 2017) hold great promise for enhanced explainability, while integrating experts in the decision making and interpretation of the XAI justification (Kieseberg et al., 2015; Kieseberg et al., 2016) may improve the clinical relevance. Additionally, implementing these methods in real-time in the clinical setting can help to improve the applicability of ML models in clinical setting (Xiang et al., 2024) Therefore, future studies should incorporate XAI methodologies to improve model transparency, elucidate prediction errors, and enhance confidence in model applications, particularly in clinical and diagnostic settings.

## 5 Conclusion and future directions

This study highlights the potential of ML-based segmentation techniques for automating plantar pressure analysis, to enable the efficient processing of large multicenter datasets across diverse hardware setups. These methods have the potential to decrease costs by reducing the reliance on extensive human labeling, thereby minimizing subjective bias through ML-driven standardization. As a practical use case, the proposed pipeline can be integrated with clustering models (Deschamps et al., 2013; Bennetts et al., 2013) to group similar pressure profiles, which can then help optimize classification models to distinguish between healthy and pathological groups (Chae et al., 2020; Han et al., 2023). This comprehensive approach can potentially support clinical workflows by providing actionable insights into foot orthopedics, that can help streamline analysis and aid in diagnostic decision-making. Nevertheless, expert reviews and adjustments in critical cases, particularly in regions such as metatarsal area 1 and interdigital space 2–3, where higher modeling errors were observed, are essential to ensure reliable and valid outcomes in critical use-case scenarios. Therefore, the findings emphasize the importance of integrating expert oversight into the system, following paradigms such as the “doctor-in-the-loop” approach (Kieseberg et al., 2015; Kieseberg et al., 2016).

## Data Availability

The raw data supporting the conclusions of this article will be made available by the authors, without undue reservation.
